# Repair of a Chronic, Traumatic Pediatric Macular Hole Using an Internal Limiting Membrane Flap and Direct Silicone Oil “Drop” Stabilization: A Case Report

**DOI:** 10.3390/reports9010030

**Published:** 2026-01-20

**Authors:** Shravan V. Savant, Neeket R. Patel, David J. Ramsey, Jeffrey Chang

**Affiliations:** 1 Department of Ophthalmology, Rutgers Robert Wood Johnson Medical School University Hospital, New Brunswick, NJ 08901, USA; 2 Department of Ophthalmology, Beth Israel Lahey Health, Burlington, MA 01805, USA; 3 Division of Ophthalmology, UMass Chan Medical School, University of Massachusetts, Burlington, MA 01805, USA; 4 Department of Ophthalmology, Tufts University School of Medicine, Boston, MA 02111, USA; 5 Department of Biomedical Sciences and Disease, New England College of Optometry Graduate Faculty, Boston, MA 02115, USA

**Keywords:** macular hole, pediatrics, ocular trauma, vitreoretinal surgery, ILM flap, silicone oil, case report

## Abstract

**Background and Clinical Significance:** Macular holes are rare in pediatric patients and most often result from blunt trauma, commonly from soccer-related injuries. These cases present unique challenges due to delayed presentation, tightly adherent hyaloid layers, and difficulties with postoperative positioning. Larger, chronic macular holes have low spontaneous closure rates and poorer surgical outcomes, necessitating advanced surgical approaches. Herein we report a case of chronic traumatic macular hole in a pediatric patient that closed with an internal limiting membrane (ILM) flap surgical technique with silicone oil tamponade. **Case Presentation:** A 15-year-old male patient presented with a history of blunt ocular trauma from a soccer ball one year prior, resulting in a large chronic macular hole. The decision was made to perform pars plana vitrectomy with an inverted ILM flap technique and silicone oil tamponade. To stabilize the ILM flap and prevent displacement, a novel technique involving the placement of a single drop of silicone oil on the retinal surface prior to complete silicone oil fill was employed. This “silicone oil drop” technique allowed for smoother propagation of the oil over the flap, effectively securing it without the need for additional manipulation or perfluorocarbon liquid. Postoperatively, the macular hole was closed, and the patient’s vision improved. **Conclusions:** This case highlights the potential benefits of the ILM flap technique in treating pediatric macular holes with utilization of silicone oil as not only a tamponade but as a method to stabilize the flap.

## 1. Introduction and Clinical Significance

Macular holes are uncommon in the pediatric age group, but when they do occur, it is most often because of trauma [1,2]. In the absence of spontaneous closure, which may take weeks or months, vitrectomy with membrane stripping is often indicated, but these surgeries are complex in pediatric eyes because of their tightly adherent hyaloid layer. In addition, children and their care givers often have difficulty following the necessary positioning in the postoperative period.

Furthermore, delayed diagnosis in children can lead to chronic, “stiff” macular holes, which tend to have lower surgical success rates [2]. In such cases, inner limiting membrane (ILM) peel alone may not be sufficient to close the macular hole. Additional techniques used for recurrent, large, or chronic macular holes such as ILM flaps, autologous retinal transplantation, and human amniotic membrane grafts have been adopted with varying rates of success [3]. These techniques may sometimes require perfluorocarbon liquid to allow for stabilization and adherence of the tissue being used over the macular hole [4]. Delayed presentation and consequent need for atypical surgical techniques for success in the treatment of pediatric macular holes prove challenging. We present a case of a large chronic macular hole being closed with an ILM flap technique and a novel silicone oil “drop” technique to stabilize the flap, followed by silicone oil tamponade. This case report has been reported in line with the SCARE checklist.

## 2. Case Presentation

**Figure 1:**
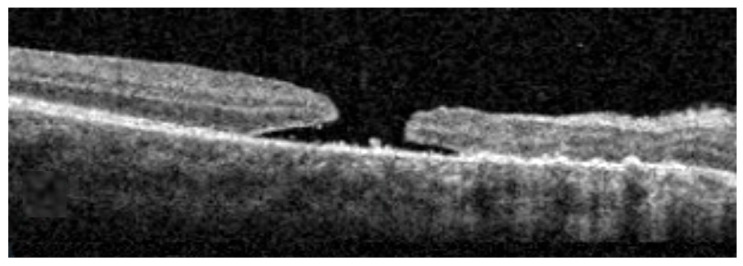
Spectral-domain OCT macular scan at initial evaluation (Cirrus HD-OCT, Carl Zeiss Meditec, Inc. Jena, Germany). Scale bar 500 µm.

**Figure 2:**
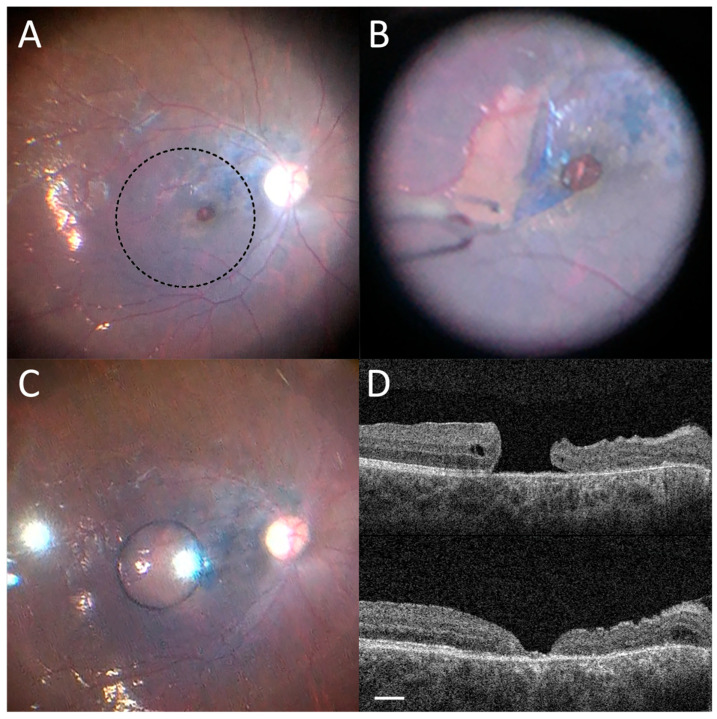
(**A**) Brilliant blue dye staining of ILM. (**B**) Inverse temporal flap creation with ILM forceps. (**C**) Single silicone oil drop placed over the macula temporal to the flap. (**D**) Resolution of macular hole after silicone oil removal. Top Panel: Spectral-domain OCT macular scan of the macula one day prior to surgery. Bottom Panel: Spectral-domain OCT macular scan of the macula on post-operative day one demonstrating closure of the macular hole. Scale bar 500 µm.

A 15-year-old Black male with no medical or prior ophthalmic history presented to the retina clinic with sudden vision loss in the right eye after blunt trauma caused by a soccer ball three days prior. Visual acuity was count fingers at 2 inches in the right eye and 20/20 in the left eye. The pupils were symmetric with no afferent pupillary defects and normal anterior segment examination bilaterally. Intraocular pressure was 19 mmHg in the right eye and 20 mmHg in the left eye and there were no signs of angle recession on gonioscopy. Fundus exam of the right eye revealed a vitreous hemorrhage and commotio retinae without retinal breaks. The commotio retinae was limited to the supertemporal quadrant with mild intraretinal hemorrhages and overlying mild–moderate peripheral vitreous hemorrhage overlying the area. A large macular hole was evident. Optical coherence tomography (OCT) imaging of the macula identified a full-thickness macular hole measuring 610 µm in horizontal diameter (Figure 1). Dilated exam of the left eye was unremarkable. In the setting of recent trauma, observation was recommended with planned reassessment in 1 month. Unfortunately, the patient was lost to follow-up. Follow-up exam one year later revealed subjectively stable but poor vision, measured as count finger vision at 1 foot with a macular hole, which had increased in size on OCT imaging to 800 µm, now associated with cystic macular edema (Figure 2D, upper panel). After consulting with the patient’s mother, she provided formal written consent for the procedure. A macular hole repair by means of 25-gauge vitrectomy with ILM flap and silicone oil tamponade was scheduled. As the patient traveled internationally for eye care, the decision to use silicone oil was made primarily because of the patient’s desire to travel home by air sooner after the surgery.

The surgery was performed under monitored anesthesia care with intravenous sedation and a peribulbar block of Bupivacaine 0.75% and hyaluronidase. Less than 2 mg of washed triamcinolone acetonide (40 mg/mL) was instilled into the eye to stain the hyaloid and ensure adequate posterior vitreous detachment induction. Brilliant blue G dye (TissueBlue^®^, 0.025% ophthalmic solution; Dutch Ophthalmic USA, Exeter, NH, USA) was used to stain the ILM and an inverse temporal flap was created using ILM forceps (GRIESHABER® REVOLUTION® DSP ILM Forceps, 25+ gauge; Alcon Grieshaber AG, Schaffhausen, Switzerland; Figure 2A,B). An air fluid exchange was performed with nasal drainage at the disc allowing for ILM to flap over the macular hole. Under direct visualization, a single silicone oil drop was placed over the macula temporal to the flap (Figure 2C). As the oil drop propagated nasally and coated the macula, the flap was secured by the weight of the overlying oil and was stabilized over the macular hole due to the oil’s surface tension. Silicone oil fill was then completed. On post-op day one, the macular hole was closed with stable count finger vision in the eye (Figure 2D, bottom panel). Removal of silicone oil was performed at post-op month 5 and the patient’s vision improved to 20/100. The macular hole remained closed.

## 3. Discussion

It is common for pediatric retinal pathology to present in a delayed, chronic fashion because children are often unable to adequately describe or understand the meaning of visual symptoms or seek eye care on their own, and may not always have an experienced healthcare advocate in the form of a parent or other guardian. This impacts the surgical success rate, which is significantly lower when compared with more acute macular holes [5]. Pediatric macular holes are most commonly caused by blunt trauma, especially from soccer-ball related injuries, which have been described in several studies [2,6]. Notably, Helmy et al.’s extensive and recent systematic review of traumatic macular holes reported that injuries linked to soccer were the most common cause of such pathology in children and adolescents [7]. While the overall incidence of traumatic macular holes is low, the risk of this and other visually debilitating conditions justifies the recommendation of eye protection in sport.

While our patient ended up requiring surgical intervention, studies have shown that spontaneous traumatic macular hole recovery can occur in up to 25% of cases within the first three to six months, with closure more common in patients who are younger than 18 years of age or with small macular holes (diameters less than 200 µm) [1,3,5,8,9]. A large prospective study by Chen et al. showed no difference in foveal microstructure and final visual acuity between eyes observed for six months with spontaneous closure of the macular hole compared to eyes taken for immediate surgery, supporting the role of observation in the acute post-injury period [10]. As 80% of spontaneous hole closures occurred within the first three months of observation, it is therefore reasonable to consider three months as an adequate initial window of time to observe prior to surgical planning. Longer periods of observation may contribute to surgical complexity and permit atrophy of retinal tissue to progress, contributing to worse surgical and visual outcomes.

There is no consensus on the surgical approach of pediatric macular holes. The majority of previously published cases discuss standard vitrectomy with posterior vitreous detachment induction with ILM peeling [6,7]. Additional adjuvant therapies such as platelet concentrates and recombinant growth factors have been reported with high success rates. There have been little to no publications on the utility of procedures such as ILM flaps in pediatric macular hole repair. Kothari et al. had a multi-center study of outcomes for pediatric patients and revealed that all patients who underwent macular hole repair with an average minimal length diameter below 384 µm had a success rate of 81% with primary vitrectomy and up to 94% with a second procedure. However, in all 31 of these cases, no primary or secondary procedure included the use of an ILM flap [6]. Given that our patient’s macular hole was on the larger end of the spectrum with cystic intraretinal edema, we felt an ILM flap provided the highest chance of single-surgery success. Most of the literature regarding the superiority of ILM flaps in large holes comes from the adult literature, with the CLOSE Study Group (Classification for Large Macular Hole Studies) being one of the largest groups to classify and determine surgical outcomes of macular holes based on the size of the minimal length diameter. Under their classification, our patient would qualify as an extra-extra-large or “XXL” macular hole (minimal linear diameter > 800 µm but less than 1000 µm), which with an ILM flap has a closure rate of 92% (compared to 80% closure rate in ILM peeling alone) and approximate average of −0.2 increase in visual acuity. Other reported uses of ILM flap in pediatric macular holes utilized adjuvant autologous plasma concentrate or were of smaller minimum length diameters [11,12,13]. Our patient introduces the idea of successful visual rehabilitation from the ILM flap and may be beneficial to consider in these pediatric patients where the visual prognosis may be guarded.

Silicone oil as a tamponade was advantageous in this pediatric case for two main reasons. First, the patient had to travel by air, precluding the use of intraocular gas. Second, this tamponade agent protected against the need for positioning, which can be difficult to enforce in children, leading to surgical failure. Notably, in this case, the silicone oil provided an additional benefit of stabilizing the ILM flap. The utility of silicone oil as an adequate tamponade comes from its ability to prevent intraocular fluid from migrating into the subretinal space. This is because of the high interfacial tension between silicone oil and water (44 dynes/cm). In addition, its higher shear viscosity reduces movement of the silicone oil bubble within the eye and prevents hydraulic-based migration of aqueous humor through retinal breaks into the subretinal space [14]. The first oil droplet placed on the retinal surface was able to propagate along the plane of the temporal flap nasally, and by exerting surface tension prevented any residual fluid from the air fluid exchange from dislodging the flap. Furthermore, residual fluid would be unable to displace the oil droplet due to its high sheer viscosity. This stabilization obviated the need for another intraocular tamponade such as perfluorocarbon heavy liquid. The use of perfluorocarbon heavy liquid to stabilize the ILM flap could serve as an alternative approach, albeit one that has not been trialed in our practice. Moreover, silicone oil tamponade does require a second surgery and does have added risks, such as secondary glaucoma, band keratopathy, and rare unexplained central vision loss [15,16]. To prevent the need for silicone oil and to mitigate the concern for post-operative positioning, newer techniques such as superiorly hinged ILM flap have been developed and may have added benefits in the pediatric population [17,18]. In our case, due to retinal thinning seen in the superonasal and inferonasal macula on OCT from the initial trauma, we preferred to avoid manipulating the ILM over this atrophic area and initiated the ILM flap from the relatively normal temporal macula.

It is important to note we present a single case, which limits the generalizability of our technique. However, we believe the ability of silicone oil to provide adequate intraoperative tamponade, act as a stabilizer of an ILM flap, and prevent intraocular fluid from migrating into the subretinal space makes this a more forgiving post-operative tamponade agent, and our drop technique is especially likely to benefit pediatric patients treated for large traumatic macular holes. Indications favoring this approach include pediatric patients, chronic macular holes, or need for travel via air or at high altitudes. The unique nature of our case and limited literature available make it difficult to compare our technique directly to the previously mentioned alternative surgical techniques utilized in the treatment of pediatric macular holes. Further reports are needed to draw any definitive conclusions.

## 4. Conclusions

In pediatric traumatic macular holes, while short-term observation should be pursued to allow time for spontaneous closure, surgical intervention is often needed to achieve anatomic and visual recovery. To close large macular holes, ILM flap techniques have been described at length in the adult population [8]. Our case illustrates how this advanced surgical intervention may also benefit pediatric cases. While silicone oil tamponade poses both risks and benefits, when required, the placement of an initial drop of oil on the surface of the retina, proximal to the flap, may allow for smoother propagation of the medium over the flap, thereby aiding stabilization of the tissue complex without the need for further manipulation with other agents or techniques.

## Data Availability

Supporting data can be found and is referenced within the publication. No other new data were created.

## Abbreviations

The following abbreviations are used in this manuscript:

OCT	Optical Coherence Tomography
CLOSE	Study Group (Classification for Large Macular Hole Studies)
ILM	Internal Limiting Membrane
